# Supplementary material for: Adipose Tissue-Derived Mesenchymal Stem Cells Extend the Lifespan and Enhance Liver Function in Hepatocyte Organoids
Source: Int J Mol Sci. 2023 Oct 21;24(20):15429. doi: 10.3390/ijms242015429 (PMC10607770; doi:10.3390/ijms242015429)
Supplement: Supplementary file 1 [file ijms-24-15429-s001.zip › Supplemetary Figures Legends.pdf]

SUPPLEMENTARY FIGURE LEGENDS

Supplementary Figure S1. Survival of freeze-thawed primary hepatocytes (HAs) treated with 36% Percoll solution

(A) Survival rates of cells separated from the pellet (dark gray bar) and supernatant (light gray bar) after treatment with 36% Percoll solution. Open bars represent cells that were washed once with the culture medium after thawing frozen HA as a control. Error bars represent the mean ± standard deviation (SD). Different letters above each bar indicate statistically significant differences ($p < 0.05$; n = 3). (B) Expression levels of genes involved in apoptosis, including BAK1, BCL2L1, and CASP8, were analyzed using quantitative real-time PCR. Error bars represent minimum (min) and maximum (max) relative quantification (RQ) levels around mean RQ expression levels. Different letters indicate significant differences among the groups (p < 0.05, n = 5).

Supplementary Figure S2. Characterization of adipose tissue-derived mesenchymal stem cells (A-MSCs) isolated from GFP-labeled miniature pig adipose tissues

(A) Cells were subjected to differentiation in specific differentiation media for adipocytes, osteocytes, and chondrocytes for 21 d. GFP, bright field (BF), and Merge refer to the fusion of two im-ages: the expression of green fluorescent protein (GFP) under a fluorescent microscope and a BF image. (B) Differentiated cells were stained with Oil Red O, Alizarin Red S, and Alcian Blue to visualize their morphology. Macroscopic and microscopic images were acquired. Scale bars rep-resent 50 μm. (C) The expression of adipocyte-, osteocyte-, and chondrocyte-specific genes in differentiated cells was assessed using quantitative real-time PCR. This included: (i) Adipogene-sis-related genes (PPARγ and LDLR), (ii) Osteogenesis-related genes (RUNX2 and ALPL), (iii) Chondrogenesis-related genes (SOX9 and ACAN). Error bars represent minimum (min) and maximum (max) relative quantification (RQ) levels around mean RQ expression levels. Statistical significance analysis between groups was performed using the Student's t-test with SPSS 25 (n = 5). The asterisk (*) above each bar represents a statistically significant difference ($p < 0.05$; n=5). Open bars represent cells before differentiation and gray bars represent cells after differentiation.

Supplementary Figure S3. Primary hepatocytes (HAs) co-cultured with A-MSCs in 2D culture

(A) HAs were initially seeded either alone (20,000 cells/well) or co-cultured with A-MSCs (20,000 hepatocytes/4,000 A-MSCs/well) in a 24-well plate and cultured for 14 d, denoted as passage 0 (p0). Serial passaging was performed at a 1:2 ratio every 14 d. The green color under the fluorescent microscope indicates A-MSCs expressing GFP with scale bars representing 200 μm. (B) The morphology of cells at passage three is presented, with scale bars representing 200 μm.

Supplementary Figure S4. Expression of liver-specific proteins in hepatocyte organoids (HOs) co-cultured with adipose tissue derived mesenchymal stem cells (A-MSCs)

Expression of albumin (purple), cytokeratin 19 (purple), and E-cadherin 1 (green) in HOs cultured alone (A) or co-cultured with A-MSCs (B) for 14 d in vitro at passage 0. A-MSCs were not detected in HOs alone, as confirmed by GFP expression. DAPI and BF indicate nuclear and bright-field staining, respectively. all scar bars = 50 um.

Supplementary Figure S5. Functional liver assay in hepatocyte organoids (HOs) co-cultured with adipose tissue-derived mesenchymal stem cells (A-MSCs)

HOs were cultured alone (A) and co-cultured with A-MSCs (B) for 14 d in vitro at passage 0. The assays conducted included (i) Oil Red O staining for nitroglycerin and cholesterol esters, (ii) Periodic Acid Schiff (PAS) staining for glycogen, (iii) Dil Acetylated-Low Density Lipoprotein (Dil-Ac-LDL) assay for low-density lipoprotein, and (vi) Indocyanine Green (ICG) clearance assay. Scale bars represent 50 μm (n = 5).
