# Supplementary material for: Adipose Tissue-Derived Mesenchymal Stem Cells Extend the Lifespan and Enhance Liver Function in Hepatocyte Organoids
Source: Int J Mol Sci. 2023 Oct 21;24(20):15429. doi: 10.3390/ijms242015429 (PMC10607770; doi:10.3390/ijms242015429)
Supplement: Supplementary file 1 [file ijms-24-15429-s001.zip › Supplemetary Methods.pdf]

SUPPLEMENTAL METHODS

Confirmation of A-MSCs

A-MSCs derived from the adipose tissue of GFP transgenic pigs were cultured in adipogenic, osteogenic, and chondrogenic induction media for three weeks. Tis-sue-specific staining, including Oil Red O, Alizarin Red S, and Alcian blue staining, was performed to assess adipocyte, osteocyte, and chondrocyte differentiation, respectively [16]. Additionally, the expression levels of tissue-specific genes, such as PPARG/LDLR for adipocytes, Sox9/ACAN for chondrocytes, and RUNX2/ALPL for os-teocytes, were analyzed using quantitative real-time PCR.
