# Supplementary material for: Adipose Tissue-Derived Mesenchymal Stem Cells Extend the Lifespan and Enhance Liver Function in Hepatocyte Organoids
Source: Int J Mol Sci. 2023 Oct 21;24(20):15429. doi: 10.3390/ijms242015429 (PMC10607770; doi:10.3390/ijms242015429)
Supplement: Supplementary file 1 [file ijms-24-15429-s001.zip › Supplemetary Tables S1-S2.pdf]

Supplementary Table S1. List of antibodies used for immunofluorescence staining.

Protein	Clonalities	Antibodies		Company	Cat. No	Dilution factor
**Albumin**	Polyclonal	1st	Anti-Albumin antibody	Abcam	ab112980	1:100
	Monoclonal	2nd	Mouse anti-goat IgG-TR	Santacruz	sc-3916	1:200
**Cytokeratin 19**	Polyclonal	1st	Anti-cytokeratin 19	Abcam	ab84632	1:200
	Polyclonal	2nd	Goat anti rabbit IgG H&L (AlexFluor 555)		ab150078	1:200
**E-Cadherin 1**	Polyclonal	1st	E-cadherin (N-20)	Santacruz	sc-1500	1:50
	Polyclonal	2nd	Donkey anti-goat lg-FITC		sc-2024	1:100

Supplementary Table S2. Porcine primer sequences used for real-time RT-PCR.

Gene	Primer sequences	Product size (bp)	GenBank accession number
Albumin (ALB)	F - GAAGACACCAGTGAGCGAAAAA<br>R - AAGCAAGGCCGTCTGTTCA	69	NM_001005208.1
Transferrin (TF)	F - CCGAAGACTGTATCGCCAAGA<br>R - TAGCCTCCATCCAAGCTCATG	64	X12386.1
Alpha fetoprotein (AFP)	F - CGTGCCTCCGCCATTC<br>R - CTGAGCTTGGCACAGATCCTT	61	NM_214317.1
Serpin family A member 1 (SERPINA, AAT)	F - CCCAACCTGGCCGACTT<br>R - AAGATGTTGCTGGTATTGGACTGA	71	NM_214395.2
Cadherin 1 (CDH1)	F - CTCGTAATGATGTGGCACCAA<br>R - AAAGTTTCCAATTTCATCAGGATTG	83	NM_001163060.1
Cytochrome P450 family 1 subfamily A member 2 (CYP1A2)	F - GGCAGGGCGACGATTTC<br>R - TCAGTGACCAGAGTGAAGCTGTAGA	58	NM_001159614.1
Cytochrome P450 family 3 subfamily A member 29 (CYP3A29)	F - AGGAGGAGATTGAGGCAACTTTC<br>R - TCTGTGCCAGGGCATCGTA	61	NM_214423.1
Keratin 19 (KRT19)	F - GCAGATCAGCAAGACGGAAGT<br>R - GGCTTTCATGCTGAGCTGAGA	88	XM_003131437.4
SRY-box transcription factor 9 (SOX9)	F - GTACCCGCACCTGCACAAC<br>R - TTCAGCAGTCTCCAGAGTTTGC	63	NM_213843.2
Epithelial cell adhesion molecule (EPCAM)	F - AACCAGAGAATGCTATCCAGAACAAC<br>R - ACCCCAGCAGTGTTCACACA	124	NM_214419.1
Low density lipoprotein receptor (LDLR)	F - GCTGGACCTAACTTCGAGTATGC<br>R - TGCGTCGTCGGGAGAAA	56	AY559453.1
Peroxisome proliferator activated receptor gamma (PPARγ)	F - ACACCGAGATGCCGTTTTG<br>R - CGACAGGTCCACAGAGCTGAT	55	NM_214379.1
Aggrecan (ACAN)	F - CCGGAAGCAGCAGTCACA<br>R - TTCAAGCCAATCCACTGGTAGTC	96	NM_001164652.1
RUNX family transcription factor 2 (RUNX2)	F - CAAACAGCCTCTTCAGCACAGT<br>R - GTGCTCGGATCCCAAAAGAA	61	XM_003482203.4
Telomerase reverse transcriptase (TERT)	F - CAC TGC GAC TAT GCC AGT TAC G<br>R - GCC CTG GTT GAA GGT GAG ACT	63	AY785158.1
Tumor protein p53 (TP53)	F - AAG CCA CTG GAT GGC GAG TA<br>R - GGG CAT CCT TCA GCT CCA A	100	NM_213824.3
CDKN1A cyclin dependent kinase inhibitor 1A (CDKN1A, p21)	F - CCTCCCAGGGCAGGAAA<br>R - GCGTTTGGAGTGGTAGAAATCTG	59	XM_013977858.2
Caspase 8 (CASP8)	F - GGGAAGCCCCCATCTATGA<br>R - CCCTTGACAAGCCTGAATGAA	101	NM_001031779
BCL2 antagonist/killer 1 (BAK1)	F - TCACCCTGCCCCTAGAACCT<br>R - CTGGAATTCCGAGTCGTATCG	101	XM_001928147.2
BCL2 apoptosis regulator (BCL2)	F - GGTACCGGAGGGCATTCAGT<br>R - TCCCGGAAGAGTTCGTTCAA	100	NM_214285.1
Hypoxanthine phosphoribosyltransferase 1 (HPRT1)	F - CGGCTTGCTCGAGATGTGAT<br>R - AGCACACAGAGGGCTACGATGT	62	NM_001032376.2
